# Exploration of time–frequency reassignment and homologous inter-hemispheric asymmetry analysis of MCI–AD brain activity

**DOI:** 10.1186/s12868-019-0519-3

**Published:** 2019-07-31

**Authors:** T. Nimmy John, Puthankattil Subha Dharmapalan, N. Ramshekhar Menon

**Affiliations:** 1 Department of Electrical Engineering, National Institute of Technology Calicut, Calicut, Kerala India; 2 Department of Neurology, Sree Chitra Tirunal Institute for Medical Sciences and Technology, Trivandrum, Kerala India

**Keywords:** Alzheimer’s disease, Electroencephalography, Synchrosqueezing transform, Approximate entropy, Fractal dimension, HArS

## Abstract

**Background:**

In this study, nonlinear based time–frequency (TF) and time domain investigations are employed for the analysis of electroencephalogram (EEG) signals of mild cognitive impairment–Alzheimer’s disease (MCI–AD) patients and healthy controls. This study attempts to comprehend the cognitive decline of MCI–AD under both resting and cognitive task conditions.

**Results:**

Wavelet-based synchrosqueezing transform (SST) alleviates the smearing of energy observed in the spectrogram around the central frequencies in short-time Fourier transform (STFT), and continuous wavelet transform (CWT). A precise TF representation is assured due to the reassignment of scale variable to the frequency variable. It is discerned from the studies of time domain measures encompassing fractal dimension (FD) and approximate entropy (ApEn), that the parietal lobe is the most affected in MCI–AD under both resting and cognitive states. Alterations in asymmetry in the brain hemispheres are analysed using the homologous areas inter-hemispheric symmetry (HArS).

**Conclusion:**

Time and time–frequency domain analysis of EEG signals have been used for distinguishing various brain states. Therefore, EEG analysis is highly useful for the screening of AD in its prodromal phase.

## Background

Alzheimer’s disease (AD), a progressive brain disorder, considered to be in the category of dementia, generally occurs in the latter part of life. Degeneration of brain cells and the incidence of senile plaques are some of the changes found in the brain of AD patients [1]. It was reported by Brookmeyer et al. that by the year 2050, the total worldwide AD population would be 106.2 million with the chance of one in 85 persons suffering from AD. Approximately 48% of AD cases would be in Asia, with the percentage rising to 59% by 2050 [2]. It was also reported that ‘the community whose damage to the brain cells can’t be reversed is likely to rise to 152 million by 2050’ [3].

EEG represents the electrical brain activity, measured using electrodes placed on the scalp of the human brain. EEG signals could easily characterise clinical manifestations of various neurological disorders. Hence, EEG can be used efficiently as a diagnostic tool. Changes in EEG time series are concomitant with varying dynamics of brain function. EEG signals are used for the analysis of pathological conditions such as mild cognitive impairment [4], Schizophrenia [5, 6], Parkinson’s disease (PD) [7, 8] and Epileptic Seizure detection [9, 10]. Signal-processing techniques are used for the feature extraction and characterisation of EEG signals of various brain diseases.

Neuronal interactions occurring at various levels of temporal and spatial scales have nonlinear behaviour. The concept of nonlinear dynamics is widely used in the analysis of time series obtained from the human brain. The dynamics of the brain are comprehended using EEG signals. Hence, nonlinear methods are suitable for the analysis of EEG signals [11–13]. Both time domain and TF domain methods are employed for extracting reliable information from EEG signals of MCI–AD patients for further characterisation. The performance of the brain is evaluated under resting and cognitive states using the nonlinear time domain measures of FD and ApEn. Time-varying spectral properties of MCI–AD are presented lucidly using a time–frequency reassignment (TFR) method, SST.

Time–frequency representations (TFRs) are a graphical display that enable easy signal interpretation, analysis, detection of time and frequency information. STFT and WT are some of the techniques used to analyse non-stationary signals in both the domains of time and frequency. STFT based on fast Fourier transforms (FFT), was widely used earlier for the analysis of the signal in time and frequency domain. The spectral estimates of the mean and relative power in higher frequency bands of the EEG signal are studied using varying configurations of STFT [14]. STFT was used to obtain the spectrogram of different channels of EEG recordings from AD. The extraction of EEG features and the generation of classification models for the diagnosis of AD were reported by Podgorelec [15]. STFT extracted spectrogram features were employed in the classification of EEG signals [16].

STFT fails to represent both time and frequency information of EEG signal simultaneously. The TF trade-off that occurred in the STFT is overcome by WT. WT offered better spectral features than FFT and was well recognized for the detection of brain diseases [17]. Decision tree algorithm was employed to identify the best discriminating feature of AD from DWT decomposed EEG signals obtained under active and resting states [18]. Decision tree classifier could effectively classify MCI, AD and normal control with higher accuracy, using wavelet features than Fourier based features [19].

Nonlinear TF methods are found to be appropriate for the analysis of time series. STFT and CWT have played an imperative role in the fields of engineering and sciences. The smearing of the components usually impede the interpretations of spectral decomposition. A highly localised technique SST, overcome the drawbacks caused by STFT and CWT with the estimation of instantaneous frequency and frequency reassignment property. Instantaneous frequency can be estimated from the modulus of TF representation employing SST. SST not only provides spectral analysis but also decomposes a signal with higher precision in time and frequency [20]. Reassignment techniques sharpen the TF representation even in the multi-component signal and retain temporal localisation [21]. SST is applicable for the quantification of dynamical features of respiratory and EEG signal [22], for the detection of sleep spindles [23] and sleep stage visualisation and prediction algorithm [24].

Several nonlinear measures are used for evaluating the complexity of bio-signals. Nonlinear EEG signal analysis has been used to distinguish the various states of the brain. The irregularity associated with the time series is quantified using ApEn. Reduced values of ApEn, largest lyapunov exponent (LLE) and correlation dimension (D2) were reported for controls subjected to sound/reflexologic stimulation [25]. Abásolo et al. reported considerably lower ApEn values for AD patients [26] in the parietal region [27]. A decline in EEG irregularity was observed for AD patients using ApEn and auto mutual information (AMI). The low values revealed the dysfunction among different regions of the brain during Alzheimer’s [28, 29]. Slowing and complexity lowering of EEG signals were reported in MCI and mild AD patients, compared with healthy controls [30].

Occipital EEG changes were quantified using FD and highly reduced FD values for the autopsy-confirmed AD than probable AD group [31]. A relation between EEG bandpower and diminished values of FD has been reported for AD [32]. FD analysis using quantitative EEG with classification algorithms could effectively categorise AD and normal aging [33]. Reductions in the value of FD in AD patients confirmed the deterioration of the dimensional complexity of brain activity [34]. FD of cerebral cortical ribbon was considered as a biomarker of cerebral cortex structure in the mild AD [35]. A combination of FD calculations with entropy analysis of 3D brain scans was utilized for AD diagnosis [36]. Speech markers resulting from spontaneous speech (SS) were helpful for the early diagnosis of AD [37]. FD, LZC and Tsallis entropy (TsEn) values computed for different frequency bands revealed lower values for AD subjects than for healthy controls [38].

Numerous studies have been reported on the hemispherical asymmetry of brain activity during aging. Inter-hemispheric differences were reported to be higher in the right hemisphere than in the left during resting state, with eyes closed (awake) when compared to various sleep stages [39]. Only a limited number of studies have emerged in the analysis of inter-hemispheric asymmetry, using EEG signal. Studies were carried out to analyse the correlation of psychological pain in adults with depression using calculations of Frontal fractal dimension asymmetry (FFDA) and frontal alpha asymmetry [40]. Significant reduction in FD was reported in elderly subjects at central-parietal regions in the right brain hemisphere, compared with the left hemisphere [41].

EEG signal analysis is applied to identify various brain dynamics of MCI–AD subjects. Most of the earlier studies concentrated on either one or two mental conditions, whereas the present study comprises four protocols of eyes open (EO), eyes closed (EC) classified under resting states and MAEO (mental arithmetic eyes open) and MAEC (mental arithmetic eyes closed) classified under cognitive states. The current study proposes TF analysis on the EEG signals of MCI–AD patients using SST, CWT and STFT. The energy distribution of the MCI–AD EEG signal in the TF plane can be displayed using SST. The blurring effect of energy concentration should be reduced for the proper TF representation of MCI–AD EEG signal. The present work also attempts to depict time domain measures using FD and ApEn to characterise EEG signals of MCI–AD subjects. The nonlinear time domain features are extracted from healthy and MCI–AD to examine the cognitive decline occurring in specific lobes of the brain. Asymmetry alterations in the MCI–AD brain hemispheres were analysed using HArS. Using the time domain analysis, we aspired to test the hypotheses that (i) cognitive decline in MCI–AD is higher under cognitive task condition (ii) impairment is conspicuous in parietal lobe in MCI–AD under cognitive states (iii) asymmetric alterations exist in the right and left hemispheres of the brain in MCI–AD.

## Results

### STFT, CWT and SST

Precise representation of the energy concentration of different frequency bands of EEG signals of MCI–AD patients and controls were carried out using STFT, CWT, and SST. Time–frequency representations (TFRs) aid in the identification of signal features such as the signal component presented and the energy concentration. STFT and WT fall under the category of linear TF distributions, while SST is a nonlinear TF method. TF methods can detect the oscillatory signals with time-varying amplitude and frequency. Six-level multi-resolution decomposition using db10 was adopted for the extraction of different frequency bands of the EEG signal. db10 showed maximum correlation coefficient with the EEG signals of MCI–AD. The three time–frequency methods were employed on the wavelet decomposed levels of delta (A6)-(0–3.125); theta (D6)-(3.125–6.25); alpha (D5)-(6.25–12.5); beta (D4)-(12.5–25) and gamma (D3)-(25–50). The analysis had been carried out on EEG signals acquired from MCI–AD patients and healthy controls at different electrode locations. Various wavelet filters such as Bump, Gauss, Morlet and Mhat were used for SST analysis, of which Morlet Wavelet filter was used for further analysis. Morlet wavelet enabled us to gain insight into both time and frequency contents of the energy concentration in specific frequency bands.

**Fig. 1:**
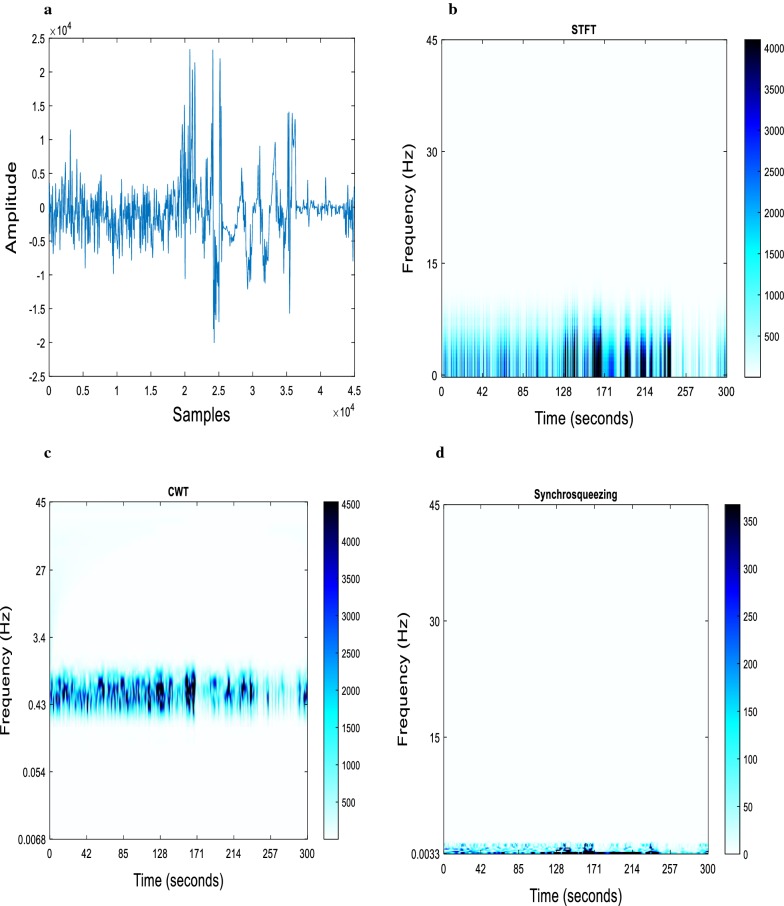
Comparison of energy concentration in delta band (0–3.125 Hz) EEG signal acquired from the parietal location (P3) for an MCI–AD patient under resting protocol **a** EEG signal, **b** STFT of **a**, **c** CWT of **a** and **d** SST of **a**. The colour bar represents the energy distribution

Figure 1 displays the energy concentration in the delta band (0–3.125 Hz) of the EEG signal acquired from the parietal (P3) location for an MCI–AD patient using STFT, CWT and SST. The STFT and CWT plots ‘smear’ the energy content of the signal resulting in decreased clarity of the frequency content presented. Gaussian window with length 256 and overlap of length 255 was used for STFT. SST plot gave a better frequency localisation so that the exact frequency information could be identified from the spectrogram analysis. Specific energy concentration at particular time duration was obtained at different frequency bands of α, β, γ and θ for signals of healthy subjects and MCI–AD patients.

STFT considers a signal stationary over the window and maps the signal to TF plane. The short window of STFT produces smearing in the frequency direction, whereas the use of the long window produces spreading in the time direction. STFT failed to achieve proper localisation in time and frequency domains according to the Heisenberg uncertainty principle. The principle states that a signal cannot be localised with high precision in both time and frequency [42, 43].

SST, a nonlinear TF technique, allows decoupling the spectra of the oscillatory components from EEG and remains stable despite errors in the signal. SST is an adaptive and invertible TF tool designed for the extraction and comparison of oscillatory components. SST provides precise frequency representation of the signal through mode decomposition with time-varying oscillatory characteristics. Compared to linear transforms, SST plots concentrate on the frequency components presented in the signal with reasonable accuracy [23, 44]. STFT plot of MCI–AD subjects displayed a spread out in low to high frequency contents. CWT based TF plot concentrates frequency components in the time-scale (TS) plane. SST reassigns scale variable of the wavelet to frequency, improves the TF representation of the signal [45]. TF analysis using SST improved the energy representation of MCI–AD subjects compared with those in STFT and CWT.

### Nonlinear parameters

The nonlinear parameters of FD and ApEn were calculated for the EEG signals acquired from the 23 electrode positions of MCI–AD and healthy controls. The parameters were calculated both for patients and controls at various lobes under resting and cognitive task conditions. The brain activity decline was correlated with the signal complexity using nonlinear measures of ApEn and FD for patients. The presence of asymmetry in homologous brain regions of the two brain hemispheres was ascertained using the computed values of FD.

### Fractal dimension

**Fig. 2:**
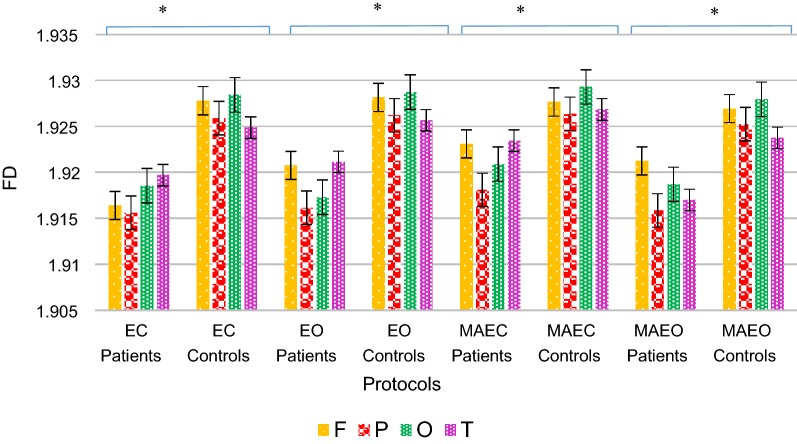
FD values of MCI–AD patients and healthy controls at different lobes under various recording protocols, the *vertical bar* represents the standard error, *p < 0.05

In this analysis, FD is used for measuring the complexity of electrical brain activity in MCI–AD patients and healthy controls. FD is based on a nonlinear dynamical system theory that captures nonlinear changes inherent in signal amplitude and frequency [46, 47]. FD is associated with a healthier or more adaptive system [48, 49]. Comparison of FD values obtained for patients and controls at different lobes under various recording protocols are demonstrated in Fig. 2.

FD values for patients were higher under MAEC protocol in comparison with other protocols. The analysis was carried out on various lobes under EO, EC, MAEO and MAEC protocols. The cognitive decline in MAEO was higher than that in MAEC, and it was higher in EC than in EO condition. The maximum value of FD for cognitive task EC protocol suggested that the MAEC protocol was suitable for locating the differences of MCI–AD EEG [50]. The mental arithmetic task performed under EC condition triggers cognitive activity. An increase in the FD values proportional to the task complexity is evident in comparison with the resting state [51]. An increase in the neuronal activity under resting state EO condition would result in higher complexity values for FD. A more brain rhythmicity was observed for the cognitive task in EO state at the early stages of AD patients. The largest values of FD occurred at the temporal location, followed by frontal and occipital with the FD value being the lowest in the parietal region (T > F > O > P) for patients (Fig. 2). EEG anomalies in dementia typically reflect disturbances in parietal lobe activities [52]. For controls, the cognitive decline of MAEO was higher than MAEC, whereas it was greater in EC than in EO condition in tandem with the observations found in MCI–AD patients.

CDR is a clinical dementia rating scale, in which values of one and less are considered for the analysis. Lower FD values were perceived in cases with CDR value of one compared with those of 0.5 at the resting and cognitive states.

The average FD values calculated for patients and controls in both the right and left parts of the brain lobes under various recording protocols are shown in Table 1. FD values are higher for patients in all the lobes at the right hemisphere of the brain and lower in the left hemisphere (Table 1a). The time series irregularity was higher in the right part of the brain, indicating the possibility of larger impairment in the left hemisphere. It points to the fact that the left part underwent faster cortical worsening than the right hemisphere in patients. FD values of left and right lobes of healthy controls at distinct recording protocols are displayed in Table 1b. Higher FD values are observed for controls than for patients in various left/right lobes of different protocols which are evident from Table 1. Higher FD values are observed for controls in comparison to MCI–AD owing to the higher brain complexity for controls both at the left/right lobe locations under distinct protocols. FD values also revealed larger irregularity in the right hemisphere for healthy controls too. MCI–AD and normal controls confirmed higher FD values at the right lobes than the left with similar patterns of variation under all the recorded protocols. EEG signals of MCI–AD had almost identical characteristics with normal aging group because of the selected MCI–AD group was chosen for a CDR value less than or equal to 1.

**Table 1:** FD values of left and right lobes for (a) MCI–AD patients and (b) healthy controls under various recording protocols

Protocols	Frontal left/right		Parietal left/right		Occipital left/right		Temporal left/right	
	FL*	FR	PL*	PR*	OL*	OR*	TL*	TR
(a) MCI–AD patients								
EC	1.9102 ± 0.004	1.9226 ± 0.002	1.9107 ± 0.0	1.9205 ± 0.0	1.9121 ± 0.0	1.925 ± 0.0	1.9133 ± 0.005	1.9261 ± 0.0007
EO	1.9137 ± 0.004	1.9278 ± 0.005	1.9115 ± 0.0	1.9208 ± 0.0	1.9137 ± 0.0	1.9209 ± 0.0	1.915 ± 0.006	1.9273 ± 0.0025
MAEC	1.9167 ± 0.006	1.9295 ± 0.003	1.9167 ± 0.0	1.9195 ± 0.0	1.9148 ± 0.0	1.927 ± 0.0	1.9199 ± 0.010	1.9289 ± 0.0015
MAEO	1.9131 ± 0.003	1.9294 ± 0.003	1.9111 ± 0.0	1.9206 ± 0.0	1.9113 ± 0.0	1.9261 ± 0.0	1.9116 ± 0.008	1.9224 ± 0.0014

Protocols	Frontal left/right		Parietal left/right		Occipital left/right		Temporal left/right	
	FL	FR	PL	PR	OL	OR	TL	TR
(b) Healthy controls								
EC	1.9251 ± 0.002	1.9304 ± 0.0004	1.9246 ± 0.0	1.9271 ± 0.0	1.9261 ± 0.0	1.9306 ± 0.0	1.9233 ± 0.003	1.9264 ± 0.002
EO	1.9257 ± 0.0019	1.93052 ± 0.0001	1.9250 ± 0.0	1.9273 ± 0.0	1.9265 ± 0.0	1.9308 ± 0.0	1.9235 ± 0.004	1.9277 ± 0.002
MAEC	1.9247 ± 0.0032	1.9305 ± 0.0008	1.9251 ± 0.0	1.9275 ± 0.0	1.9277 ± 0.0	1.9308 ± 0.0	1.9237 ± 0.0038	1.9299 ± 0.003
MAEO	1.9242 ± 0.0023	1.9295 ± 0.0007	1.9240 ± 0.0	1.9264 ± 0.0	1.9257 ± 0.0	1.9300 ± 0.0	1.9224 ± 0.003	1.9250 ± 0.003

* Two-tailed, *p* < 0.05

### Homologous areas inter-hemispheric symmetry (HArS)

Analysis of region-specific alterations in healthy elderly and MCI–AD brain activity was carried out on four different brain lobes of frontal, parietal, occipital and temporal under both resting and cognitive states. HArS calculations were based on FD values computed for the right and left-brain hemisphere of controls and patients. Zero value of HArS suggests symmetry and a positive or negative value for the index corresponds to the existence of asymmetry. Higher left FD asymmetry than right indicates a positive HArS, whereas a higher right FD asymmetry than the left specifies a negative HArS value. Table 2 mentions the values of HArS computed using FD for patients. HArS results of MCI–AD patients had larger right asymmetry than the left (Table 2). The hemispherical asymmetry was inevitable in every living being. The analysis demonstrated the left half of the brain of MCI–AD was largely impaired than the right. A dominant right hemispherical asymmetry was seen both in resting and cognitive states for the controls and patients.

**Table 2:** FD HArS values of patients

Protocols	Frontal*	Parietal*	Occipital*	Temporal*
EC	− 0.00324 ± 0.0045	− 0.00256 ± 0.0008	− 0.00336 ± 0.0004	− 0.00333 ± 0.0051
EO	− 0.00367 ± 0.0061	− 0.00243 ± 0.00081	− 0.00188 ± 0.0004	− 0.0032 ± 0.0053
MAEC	− 0.00333 ± 0.0072	− 0.00073 ± 0.0009	− 0.00318 ± 0.0004	− 0.00234 ± 0.009
MAEO	− 0.00424 ± 0.0047	− 0.00248 ± 0.0006	− 0.00386 ± 0.0013	− 0.00282 ± 0.0059

* Two-tailed, *p* < 0.05

The gradual decrease of regional heterogeneity in hemispherical asymmetry was noted in healthy elderly and MCI–AD participants. Loss of asymmetry in various brain regions may lead to dampening brain function coordination, forgetfulness and difficulty in learning tasks in healthy aging. Worsening in writing, visual processing, planning distance, recognising faces, memory, as well as social and emotional activities, are some of the consequences of asymmetry loss in patients. MCI–AD had distinct structural variation and cognitive degeneration compared to healthy aging. Therefore, hemispheric asymmetry analysis using EEG signal should help in differentiating MCI–AD with healthy controls. Negative values of HArS index represented a larger decline of complexity in the left hemisphere of the brain than the right. Progression of the disease amounting to asymmetry reduction impairs the cognitive efficiency of the brain [41, 53–56]. Similar HArS patterns are identified for controls and patients because of MCI–AD, being the very early stages of AD.

**Fig. 3:**
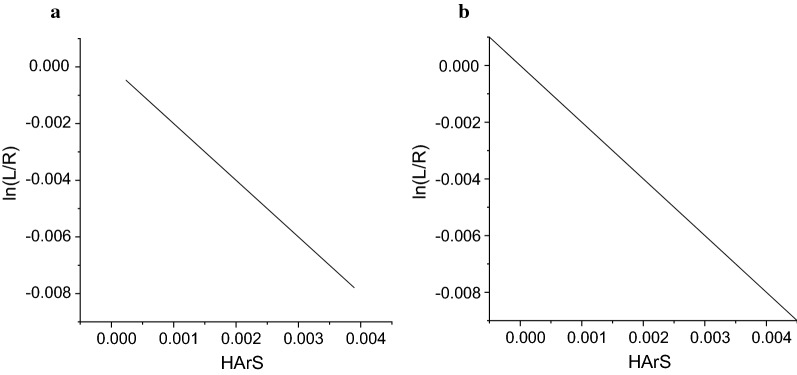
Relationship of the asymmetry metric and HArS of MCI–AD patients for calculated values of FD at **a** EC protocol, **b** MAEC protocol

The asymmetry metric is the natural log transform of left to right FD ratio and corrects overall FD asymmetry. Natural log asymmetry metric suitably summarised the asymmetrical activities of the left and right halves of the brain hemisphere. The correction was performed by the comparison of natural log metric to HArS values. HArS was correlated linearly with a natural log difference score [57, 58]. Figure 3 represents the relationship of the asymmetry metric and HArS of MCI–AD patients for EC and MAEC protocols.

Asymmetry metric score replicated differences between the asymmetrical activity of left and right hemispheres, whereas correlation reflects the similarity of asymmetry at each location. A linear relationship with the correlation between HArS and the metric score of one was observed for controls and patients for all recording protocols under resting and cognitive states. A higher score for asymmetry metric represents greater relative activity in the right hemisphere for the frontal, temporal, occipital and parietal lobes.

### Approximate entropy (ApEn)

**Fig. 4:**
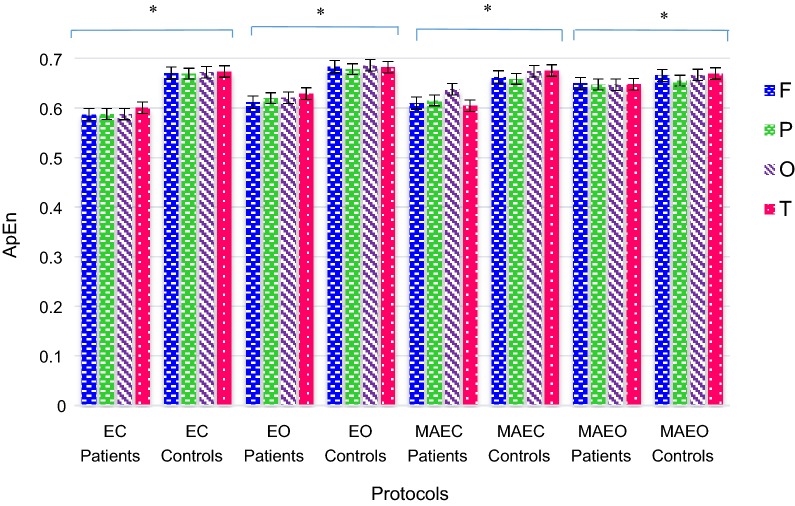
ApEn values of healthy controls and MCI–AD patients, the *vertical bar* represents the standard error, *p < 0.05

ApEn is employed as a statistic of irregularity. The lower values of ApEn indicate regularity and higher values for irregularity. Comparison of ApEn values was performed at various lobes (F, P, O, and T) under distinct recording protocols (EC, MAEC, EO and MAEO) of controls and patients (Fig. 4). ApEn values were higher for controls in all the recording protocols under both resting and cognitive task conditions. Lower values of ApEn were an indication of complexity decrease in the EEG signal acquired from MCI–AD patients. Higher brain complexity was displayed in a resting state than cognitive task condition for normal subjects. The highest ApEn values were detected at MAEO recording protocol for patients. The range of ApEn values was lower under EO and MAEC protocols from MAEO by 4–5%.

Higher values of ApEn, an indication of larger EEG complexity is observed in EO than in EC conditions, the results of which matches with the earlier studies [59–61]. The cognitive activities of the patients under MAEO were more irregular than MAEC condition, whereas EO showed a higher irregularity in comparison with EC state. Higher values of ApEn were observed for EO protocol both under resting state and under cognitive tasks in patients. The runs of a pattern of points under all intervals throughout the length of the signal remain similar for resting and cognitive task conditions during EC recording protocol.

The calculated values of ApEn were observed to be the highest for the temporal lobe and least for the frontal lobe during the resting states. Reduced ApEn values were observed for MCI–AD patients at the frontal lobe indicating complexity reduction. The lowered ApEn values were closely associated with the decline in brain activity. Impairment in both executive and attention functions, associated with the frontal lobe, had been reported in past studies in MCI–AD subjects [62, 63]. The ApEn values of controls were highest at the EO protocol followed by EC and MAEC protocol. The highest values of ApEn for controls occurred at the temporal location followed by occipital, frontal and parietal lobe in EC, MAEC and MAEO protocols. The ApEn values of healthy controls were specific to a particular region and found to be least at the parietal lobe.

The results of the FD and ApEn measures unveiled the suitability of EEG signals for distinguishing MCI–AD subjects from the healthy controls. The findings ensured that the complexity and irregularity of the EEG signal for MCI–AD patients are lowered in all the lobes under all the recording protocols. EEG analysis employing time domain measures also assist in distinguishing the patient group from healthy controls.

## Discussion

Time and time–frequency methods were employed for the EEG signal analysis of MCI–AD and healthy controls recorded under resting (EC and EO) and cognitive tasks (MAEC and MAEO). The noise elimination of EEG signal had been carried out using simultaneous low pass filtering and total variation denoising (LPF/TVD) algorithm [64]. A high value for the SNR ratio validated the performance of the denoising method adopted.

A precise time–frequency representation of MCI–AD EEG signal was developed using Synchrosqueezing transform. SST enhances the TF resolution through the computation of reassigned instantaneous frequencies. The reassignment property of SST improves the readability, which enables the facilitated spectral interpretation. Hence, highly localised TF technique of SST improves TF data of the time-varying signal. SST sharpens the time–frequency plot by concentrating the energy towards instantaneous frequency curves. SST is a reassignment vector in the frequency direction with no time shift in the TF plane. The current study focuses on the significance of SST with respect to STFT and CWT, computed on wavelet decomposed EEG signals, measured under both resting and cognitive states. The present study support the fact that the frequency representation of the EEG signal is distinguished highly by SST, with more distortion and smearing in representations of STFT and CWT [20]. The performance of CWT based SST provided better insight into the energy concentration of specific frequency bands by alleviating the blurring effect observed in STFT and CWT.

Six-level multi-resolution wavelet decomposition was carried out on the denoised signal to extract the frequency bands of δ, θ, α, β and γ. In the current analysis, SST methodology was applied to EEG frequency bands of MCI–AD and healthy controls. Reassignment procedure of SST focuses the energy of the spectrogram towards the instantaneous frequency (IF) [20]. STFT and WT decompose a signal into both time and frequency components. SST is the enhanced version of WT, integrating the components of empirical mode decomposition, using TFR algorithm [44]. The reassignment property of SST separates it from STFT and CWT and thereby provides TF localisation without time shifts. SST exemplifies a signal in TF plane instead of TS plane. The spreading of frequency components was reduced in SST compared with those in STFT and CWT.

FD analysis is used to detect the complexity of the EEG signal [65]. A reduced brain activity complexity was observed for MCI–AD patients compared with healthy controls, in line with AD studies [34]. A reduction of FD values on cortical brain regions of MCI–AD was mainly observed in frontal, occipital and parietal areas. The reduced FD values during progression of Alzheimer’s reveal neural and cognitive discrepancy in the brain region [41]. No correlation was observed between FD and CDR scales of 0.5 and 1.0 for MCI–AD patients on both cognitive and resting states. Though, for cases with CDR = 2, there is a result which contradict***s*** our observation [32]. It is inferred from the current study that FD values were lower for CDR = 1 than for CDR = 0.5 both for resting and cognitive states. Reportedly, the disorder in cognition exists in brain aging, neurodegenerative diseases and dementia [66]. Homologous inter-hemispheric asymmetry computed using the values of FD specified larger impairment in the left hemisphere of the brain in our study. Thompson et al. reported that in AD, the progression of cortical atrophy expedited in the left half of the brain than in the right part [67].

The current study also employed ApEn for the evaluation of irregularity of EEG signals in MCI–AD and controls. ApEn analysis under different protocols revealed that MCI–AD subjects had more regularity than controls in all the electrode locations. The result was consistent with the previous nonlinear studies conducted for relaxed EC state [26, 28, 68]. The dynamic brain changes resulted in aberrations in the EEG signals of MCI–AD subjects. Neurotransmitter deficiencies, losses of functional connectivity due to the death of neurons are some of the reasons responsible for such changes [11]. Larger values of ApEn for controls indicated new pattern generation in healthy controls. Deficiency of cortical activity in the cerebral cortex is due to the lack of active networks resulting in diminished ApEn values for AD patients [11]. The results suggested that there is no specific difference in the cognitive decline in MCI–AD under cognitive task compared with the resting state. The hypothesis supports parietal lobe impairment and asymmetric alterations in EEG signal at the right and left hemispheres of the MCI–AD. The findings revealed that there is loss of EEG complexity in patients at the primary phases of AD compared with healthy controls. Consequently, abnormal EEG could be used as an indicator in the early diagnosis for AD.

## Conclusion

In this paper, nonlinear methods of time–frequency and time-domain were employed to analyse EEG signals at the early stages of AD. EEG signal measurements were carried out under resting and cognitive states for MCI–AD patients and healthy controls. The nonlinear time–frequency method of SST provided spectral EEG signals with higher precision in time and frequency. SST alleviates the smearing of frequency bands and thus improves the readability of the spectrogram compared with those in STFT and CWT. The time domain measures of FD and ApEn were applied on MCI–AD and healthy EEG signals. Lower FD and ApEn values could be an indication of developing cognitive deterioration in the brain of MCI–AD patients. The time domain measures confirmed that EEG anomalies owing to dementia are reflected at the parietal lobe functions. HArS values computed from FD demonstrates a rightward hemisphere dominance. Reduction of asymmetry in the hemispheres is an indication of reduced interaction between the hemispheres. Asymmetry reduction leads to a decline in cognitive performance in patients. Cognitive worsening and memory loss are an indication of the neuronal cell death in early AD. Changes in EEG time series are concomitant with dynamics of brain function. Hence, EEG signal is a potential biomarker for the diagnosis of AD at its prodromal stages.

## Methods

### Participants

A group control study ranges from 50 to 80 years involving mild cognitive impaired subjects with MCI–AD (8 men and 7 women; age = 67.78 ± 6.10 years) and healthy controls (15 men and 12 women; age = 56.18 ± 4.78 years) were used for the analysis. Significant differences were observed for the age of patient group and controls ($F_{crit} = 34.98$, p < 0.0005). The disease severity was measured using the Clinical Dementia Rating scale (CDR). CDR for MCI–AD patients ranges from 0.5 to 1, with controls having a CDR value of 0. Controls considered for the analysis were subjects without any health issues. Mini-mental state examination (MMSE) and Addenbrooke‘s cognitive examination-revised (ACE-R) were the two neuropsychological indices used for the study. MMSE [69], a simple method out of 30, to evaluate cognition was adopted for the analysis. The patients had an MMSE score of 23.92 ± 4.15 and control group with 29.37 ± 0.92. ACE [70], a score out of 100, denotes a larger value for the better cognitive function. The MCI–AD patients who took part in this study had an ACE score of 63.85 ± 8.45 and controls with 93 ± 5.34 points. Healthy controls have an education level of 12.3 ± 3.5, and patients with 11.1 ± 3.3. The two groups are matched on educational level. Demographic details including socioeconomic strata, occupation related information are collected. Personal proforma are collected for information about the medical history. Participants with a history of any neurological/neurosurgical/psychiatric disorder are not included in the study forms the exclusion criterion. Participants are screened on the basis of MMSE and ACE-R and the diagnosis of MCI are made based upon standard criteria.

EEG data acquisition from MCI–AD patients and healthy controls were conducted at Sree Chitra Tirunal Institute for Medical Sciences and Technology, Trivandrum, Kerala, India. Ethical committee sanction was given for this work. Written informed consent was obtained from both healthy controls and caretakers of Alzheimer patients who participated in the current study.

### EEG recording

EEG was recorded simultaneously from 32 channels EEG acquisition system (NicVue, Nicolet-Viking, USA) corresponding to the International 10–20 System. The 23 channels located at frontal (Fp1, Fp2, F3, F4, F7, F8), parietal (P3, P4), occipital (O1, O2), temporal (T1, T2, T3, T4, T5, T6), C3, C4, Fz, Cz, Pz, A1 and A2 were utilized for the current analysis. Left and right earlobes (A1 and A2) were marked as reference electrodes. The sampling rate of the data recorded was 400 Hz.

EEG data was recorded for 5 min each from controls and patients under resting state and cognitive task condition. Resting EEG recordings were obtained under Eyes Open (EO) and Eyes Closed (EC) while Mental Arithmetic EO (MAEO) and Mental Arithmetic EC (MAEC) recordings were acquired for 5 min each under cognitive task condition respectively. Simple mental arithmetic exercises such as simple addition, subtraction, multiplication and division are performed during eyes open (MAEO) and eyes closed (MAEC) conditions. The artifacts in the EEG signal acquired under both resting and cognitive task conditions were removed using simultaneous low-pass filtering and total variation denoising (LPF/TVD). An improved signal-to-noise ratio on employing the denoising algorithm ensured the efficacy of the algorithm. Digitised EEG signals were analysed using MATLAB (R2017b) environment.

### Time–frequency (TF) analysis

Time–frequency analysis helps in simultaneous representations of both the time and frequency content of the signal. TF methods are useful tools for the analysis of nonstationary signals like EEG, for attaining frequency variation and energy distribution presented in the signal over time. TF methods are appropriate for the localisation of individual components presented in a multicomponent signal. Different TF methods adopted in the paper are STFT, WT and SST.

#### Short-time Fourier transform (STFT)

STFT slices the signal into small segments and applies Fourier transform (FT) on each portion. Difficulty in the selection of optimal window length of segments which contain various features and TF trade-offs are the drawbacks of STFT [15].

1 $$STFT{}_{x}^{\omega } \left( {\tau ,\omega } \right) = \int \left[ {x\left( t \right) \cdot W\left( {t - \tau } \right)} \right] \cdot e^{ - j\omega t} dt$$

where $\tau$: time parameter, $\omega$: frequency parameter, $x\left( t \right)$: signal to be analysed, $e^{ - j\omega t}$: FT Kernel (basis function), $W\left( {t - \tau } \right)$: windowing function (Analysis window).

#### Continuous wavelet transform (CWT)

Wavelet transform (WT) uses a variable window size for TF analysis. The larger time window gives good low-frequency resolution, and short time window provides good high-frequency resolution. The two types of WT available for the analysis are continuous wavelets transform (CWT), and discrete wavelets transform (DWT) [71]. CWT is represented as:

2 $$W_{s} \left( {a,b} \right) = \frac{1}{{\sqrt {\left| a \right|} }}\int s\left( t \right)\psi^{*} \left( {\frac{t - b}{a}} \right)dt$$

where $a$: scale parameter, $b$: translation parameter, $\frac{1}{\sqrt a }$: normalization constant, $s\left( t \right)$: signal to be analyzed, $W_{s} \left( {a,b} \right)$: coefficients representing concentrated time–frequency, $\psi^{*} \left( {\frac{t - b}{a}} \right)$: mother wavelet.

DWT is determined by passing the signal through a series of high and low pass filters. DWT of the signal is given by:

3 $$y_{low} \left( n \right) = \sum s\left[ k \right]g\left[ {2n - k} \right]$$

4 $$y_{high} \left( n \right) = \sum s\left[ k \right]h\left[ {2n - k} \right]$$

where $y_{low} \left( n \right)$: approximation coefficients, $y_{high} \left( n \right)$: detail coefficients, $g$: low-pass filter, $h$: high-pass filter, $s\left[ k \right]$: signal to be analysed.

CWT splits continuous time signal into wavelets, whereas DWT is the discretised version of CWT. The present work used CWT as a continuous-time signal, which is considered for the analysis.

#### Synchrosqueezing transform (SST)

SST is an invertible and adaptive transform that improves the quality of TFR by condensing it along the frequency axis. SST method is robust to noise and TF plot represents frequency information corresponding to the specific frequency bands. SST concentrates energy content to a small spectral band and is suitable for TF localisation. Mainly two types of SST methods are available: STFT-based SST and Wavelet-based SST. The present study employs wavelet-based SST as the reconstruction error in the signal gets a globally constant value. It also enables sharper spectral representation of the signal at high frequencies [72]. The detailed steps for SST calculation are outlined in Herrera et al. [44]. SST method uses mapping of time-scale (TS) plane to time–frequency (TF) plane.

5 $$SST\left( {\omega_{l} , b} \right) = \frac{1}{\Delta \omega }\mathop \sum \limits_{{a_{k: } \left| {\omega \left( {a_{k} ,b} \right) - \omega_{l} } \right| \le {\raise0.7ex\hbox{${\Delta \omega }$} \!\mathord{\left/ {\vphantom {{\Delta \omega } 2}}\right.\kern-0pt} \!\lower0.7ex\hbox{$2$}}}} W_{s} \left( {a_{k} ,b} \right)a^{ - 3/2} \Delta a_{k}$$

where $$a, b$$: discrete values, $$\Delta a_{k} = a_{k - 1} - a_{k}$$: scaling step, SST is decided at the centres of $$\omega_{l}$$, with a frequency range: $$\omega_{l} - {\raise0.7ex\hbox{${\Delta \omega }$} \!\mathord{\left/ {\vphantom {{\Delta \omega } 2}}\right.\kern-0pt} \!\lower0.7ex\hbox{$2$}},\;\;\omega_{l} + {\raise0.7ex\hbox{${\Delta \omega }$} \!\mathord{\left/ {\vphantom {{\Delta \omega } 2}}\right.\kern-0pt} \!\lower0.7ex\hbox{$2$}}$$, having $$\Delta \omega = \omega_{l} - \omega_{l - 1,}$$ $$W_{s} \left( {a_{k} ,b} \right):$$ coefficients representing concentrated time–frequency, $$\omega \left( {a_{k} ,b} \right):$$ instantaneous frequency.

The higher resolution and exact reconstruction of signal components in frequency bands of interest discriminate SST from STFT and CWT. SST is found to be producing highly precise results in the current spectral analysis.

### Fractal dimension (FD)

FD is one of the applications of chaos theory measuring the complexity of the neuronal cell profiles [73]. FD is calculated as per the algorithm proposed by Higuchi et al. [74]. Increased FD value indicates higher irregularity of the series.

Let the time series be: X = *x* [1], *x* [2],…, *x* [N]. Form *‘k’* new time series,

6 $$X_{k}^{m} = \left\{ {x\left[ m \right], \;x\left[ {m + k} \right], \;x\left[ {m + 2k} \right], \ldots ,x\left[ {m + int\left( {\frac{N - m}{k}} \right) \times k} \right]} \right\}$$

The length of the new series is given by,

7 $$\begin{aligned} L\left( {m,k} \right) & = \frac{1}{k}\left( {\mathop \sum \limits_{i = 1}^{{int\left( {\frac{N - m}{k}} \right)}} \left| { x\left[ {m + ik} \right] - x\left[ {m + \left( {i - 1} \right) \times k} \right] } \right|} \right) \\ & \quad \times \left[ {\frac{N - 1}{{\left( {int\left( {\frac{N - m}{k}} \right) \times k} \right)}}} \right]\end{aligned}$$

where $$m = 1, 2, . . ., k;$$ $$k = 1, 2, . . .,k_{max}$$.

Mean length $L\left( k \right)$ is given by:

8 $$L\left( k \right) = \frac{1}{k}\left( {\mathop \sum \limits_{m = 1}^{k} L\left( {m,k} \right)} \right)$$

FD is the slope of $ln\left[ {L\left( k \right)} \right]$ over $ln\left( {1/k} \right)$. The selection of the appropriate value for k_max_ is performed by plotting FD values against the range of k_max_. The point where FD plateaus observed is taken as the saturation point, and the value is selected as k_max_ [75]. The value of k_max_ chosen for the present study is 6.

### Homologous areas inter-hemispheric symmetry (HArS)

The symmetry in FD of homologous areas of brain hemisphere is evaluated for both MCI–AD and healthy subjects in addition to the nonlinear measure FD. The study on FD inter-hemispheric asymmetry is carried out to explore whether the nonlinear complexity measure FD could reveal asymmetry underlying homologous regions of the brain under the resting and cognitive task states. The homologous areas of brain hemispheres: Fp1–Fp2, F3–F4, P3–P4, O1–O2, T1–T2, F7–F8, T3–T4 and T5–T6 pairs are used for the analysis. Following the work of Smits et al. [41], the formula used for the calculation of FD HArS is chosen.

9 $$FD HArS = \frac{{ FD_{left\,channel} - FD_{right\,channel} }}{{FD_{left\,channel} + FD_{right\,channel} }}$$

### Asymmetry metric

Computation of asymmetry metric is convenient for assessing relations between EEG asymmetry and behavior. The asymmetry metric score is a unidimensional scale, which represents the asymmetry activity of both the left and right hemispheres. The natural log asymmetry metric is the difference between natural log-transformed scores. The subtraction of two natural-log transformed scores is equal to natural log-transform of the ratio of the scores. Log difference scores are developed to provide a single metric of asymmetry. Difference score conveniently summarises the relative activity at homologous left and right electrode positions. Asymmetry metric is given by [57, 58]:

10 $$\ln \left( L \right) - \ln \left( R \right) = { \ln } \left( {L/R} \right)$$

‘${\text{L}}$’ for left electrode locations and ‘${\text{R}}$’ for right electrode positions.

### Approximate entropy (ApEn)

ApEn is a measure of complexity and regularity of a system. Lower ApEn is the quantification of predictability, whereas higher ApEn indicates unpredictability of a time series [76]. ApEn established by Pincus is used for short data series [77, 78]. The algorithm of ApEn is performed as per the reports of Pincus et al. [79]. Let *N* point time series be *x* (1), *x* (2),*…*, *x* (N) having embedding space $R^{m}$, ApEn is defined as:

11 $$\begin{aligned} ApEn\left( {m,r,N} \right) & = \frac{1}{{\left( {N - m + 1} \right)}}\mathop \sum \limits_{i = 1}^{{\left( {N - m + 1} \right)}} logC_{i}^{m} \left( r \right) \\ & \quad - \frac{1}{{\left( {N - m} \right)}}\mathop \sum \limits_{i = 1}^{{\left( {N - m} \right)}} logC_{i}^{m + 1} \left( r \right) \end{aligned}$$

where $C_{i}^{m} \left( r \right) = \frac{1}{{\left( {N - m + 1} \right)}}\sum\nolimits_{j = 1}^{{\left( {N - m + 1} \right)}} {\left( {r - x_{i} - x_{j} } \right)}$, where *N* is the time series length, *m* is the comparing length of the sequences and *r* is the tolerance level [80]. The *‘m’,* and *‘r’* assigned for the present study are 2 and 0.2 * standard deviation of the series, respectively [79]. ‘$x_{i} - x_{j}$’ is the distance between the vectors.

### Statistical analysis

The average values of FD calculated over all the EEG electrode locations are comparatively lower in MCI–AD patients than in the healthy control group. Significant group differences in FD values are observed using Two-way ANOVA among the four different protocols of EO, EC, MAEO and MAEC in various lobes (F_crit_ = 3.86; p < 0.005) of patient and control group. Bonferroni correction gave a p-value of 0.00833. No statistically significant difference was observed in the values of HArS in both patient and control group among the recording protocols (p > 0.05). The significance of HArS values in the lobes of frontal, parietal, occipital and temporal regions in both controls and patients indicated the existence of asymmetry between the left and right hemispheres of the brain (F_crit_ = 5.98; p < 0.0002). The statistical significance of the HArS with asymmetry metric is confirmed using One-Way ANOVA (F_crit_ = 4.493998; p < 0.0001). A significant difference in ApEn values found among the recording protocols of controls and patients showed the complexity difference of MCI–AD and healthy controls (F_crit_ = 4.49; p < 0.0003). Bonferroni corrected p-value is 0.008.

## Data Availability

The data that support the findings of this study are available from Sree Chitra Tirunal Institute for Medical Sciences and Technology (SCTIMST), Trivandrum, Kerala, India, but restrictions apply to the availability of these data, which were used under license for the current study, and so are not publicly available. Data are however available from the authors upon reasonable request and with permission of SCTIMST.

## Abbreviations

TF: time–frequency
EEG: electroencephalogram
MCI–AD: mild cognitive impairment–Alzheimer’s disease
SST: synchrosqueezing transform
STFT: short-time Fourier transform
CWT: continuous wavelet transform
FD: fractal dimension
ApEn: approximate entropy
HArS: homologous areas interhemispheric symmetry
AD: Alzheimer’s disease
PD: Parkinson’s disease
TFR: time–frequency reassignment
TFRs: time–frequency representations
ME: mixture of expert
SSTIF: synchrosqueezing transform-derived instantaneous frequency
FSST: STFT based SST
WSST: CWT based SST
CEEMD: complete ensemble empirical mode decomposition
SSTEDR: synchrosqueezing transform electrocardiography derived respiration
AF: atrial fibrillation
BCI: brain–computer interface
LLE: largest Lyapunov exponent
D2: correlation dimension
AMI: auto mutual information
SS: spontaneous speech
LZC: lempel–Ziv complexity
TsEn: tsallis entropy
HAROLD: hemispheric asymmetry reduction in old adults
MRI: magnetic resonance imaging
FFDA: frontal fractal dimension asymmetry
F: frontal
P: parietal
T: temporal
O: occipital
EC: eyes closed
EO: eyes open
MAEC: mental arithmetic eyes closed
MAEO: mental arithmetic eyes open
LPF/TVD: simultaneous low pass filtering and total variation denoising
IF: instantaneous frequency
CDR: clinical dementia rating scale
ACE-R: Aaddenbrooke’s cognitive examination-revised
MMSE: Mini-mental state examination
DWT: discrete wavelet transform
TS: time-scale
FL: frontal left
FR: frontal right
PL: parietal left
PR: parietal right
OL: occipital left
OR: occipital right
TL: temporal left
TR: temporal right
